# A New Era, New Risks: The Cardio-Oncology Perspective on Immunotherapy in Non-Small Cell Lung Cancer

**DOI:** 10.3390/cancers17213443

**Published:** 2025-10-27

**Authors:** Luigi Tarantini, Giuseppina Gallucci, Alessandro Inno, Andrea Camerini, Maria Laura Canale, Mario Larocca, Francesca Zanelli, Maria Pagano, Giulia Alberti, Patrizia Ciammella, Nicola Maurea, Stefania Gori, Alessandro Navazio, Carmine Pinto

**Affiliations:** 1 Cardioncology Clinic—Cardiologia Ospedaliera AUSL—IRCCS “Tecnologie Avanzate e Modelli Assistenziali in Oncologia”, 42100 Reggio Emilia, Italy; luigi.tarantini@ausl.re.it (L.T.); alessandro.navazio@ausl.re.it (A.N.); 2 Independent Researcher, 85025 Melfi, Italy; 3 Medical Oncology, IRCCS Ospedale Sacro Cuore Don Calabria, 37024 Negrar di Valpolicella, Italy; alessandro.inno@sacrocuore.it (A.I.); stefania.gori@sacrocuore.it (S.G.); 4 Medical Oncology, Versilia Hospital, Azienda USL Toscana Nord-Ovest, 55041 Lido di Camaiore, Italy; andrea.camerini@uslnordovest.toscana.it; 5 Cardiology, Versilia Hospital, Azienda USL Toscana Nord-Ovest, 55041 Lido di Camaiore, Italy; marialaura.canale@uslnordovest.toscana.it; 6 Provincial Medical Oncology, Department of Oncology and Advanced Technologies, AUSL-IRCCS in Tecnologie Avanzate e Modelli Assistenziali in Oncologia, 42100 Reggio Emilia, Italy; mario.larocca@ausl.re.it (M.L.); francesca.zanelli@ausl.re.it (F.Z.); maria.pagano@ausl.re.it (M.P.); giulia.alberti@ausl.re.it (G.A.); carmine.pinto@ausl.re.it (C.P.); 7 Radiation Oncology Unit, Azienda USL-IRCCS di Reggio Emilia, 42100 Reggio Emilia, Italy; patrizia.ciammella@ausl.re.it; 8 Cardiology Division, Istituto Nazionale Tumori-IRCCS-Fondazione G. Pascale, 80131 Napoli, Italy; n.maurea@istitutopascale.na.it

**Keywords:** non-small cell lung cancer, cardiovascular diseas, immunotherapy, cardio-oncology, cardio-immuno-metabolic risk, cardiovascular toxicity

## Abstract

Lung cancer (LC) remains the leading cause of cancer-related mortality worldwide. In recent years, mortality rates have declined due to antismoking policies, earlier detection, and the advent of targeted therapies and immunotherapy, particularly for non-small cell lung cancer (NSCLC), which accounts for 85% of all cases. With improved survival, however, LC patients are increasingly exposed to competing causes of mortality, among which cardiovascular disease (CVD) is highly prevalent, affecting 30–50% of patients and contributing to nearly 30% of deaths. This burden reflects both shared risk factors and the cardiotoxic potential of radiotherapy, chemotherapy, and immunotherapy. Beyond acute adverse cardiovascular events during treatment, real-world data indicate that immune checkpoint inhibitors (ICIs) may also exert chronic cardiovascular effects, significantly accelerating the atherosclerotic process in multimorbid patients. These findings underscore the importance of accurate baseline assessment and aggressive management of cardiovascular risk factors in LC patients—particularly in the adjuvant and neoadjuvant settings, where longer survival is anticipated. Moreover, long-term monitoring should be implemented through a tailored, multiparametric strategy that integrates novel biomarkers and advanced artificial intelligence–assisted imaging techniques. Achieving this ambitious goal requires the close collaboration of a multidisciplinary team, with cardiologists playing a pivotal role. This review will address the complexity of LC patients, focusing on the interplay of cardio-immuno-metabolic factors, summarizing the cardiovascular impact of immunotherapy across metastatic, locally advanced, and perioperative settings, and outlining practical strategies for the management of these vulnerable patients.

## Simple Summary

Lung cancer (LC) remains the leading cause of cancer-related mortality worldwide. Mortality has recently declined, particularly for non-small cell lung cancer (NSCLC), which accounts for 85% of cases. This improvement reflects not only antismoking policies and earlier detection, but also the pivotal role of targeted therapies and immune checkpoint inhibitors in modern LC management. However, real-world observational studies in multimorbid populations have revealed emerging cardiovascular implications of immunotherapy, including potential effects on atherosclerosis, highlighting their increasing clinical significance. These findings emphasize the importance of comprehensive baseline cardiovascular risk assessment, identification of modifiable risk factors, and proactive management, particularly in the adjuvant and neoadjuvant settings where survival is longer. Furthermore, they support the implementation of structured, long-term monitoring strategies, leveraging multiparametric approaches that integrate advanced imaging modalities with prognostic biomarkers, to optimize cardiovascular outcomes and guide personalized care in this vulnerable patient population.

## 1. Introduction

Lung cancer (LC) remains the leading cause of cancer-related mortality worldwide, representing a major public health challenge. In 2022, LC accounted for 2,480,675 estimated cases and 1,817,469 deaths, approximately 18% of all cancer-related fatalities globally [1].

In recent years, LC mortality rates have declined, particularly for non-small cell lung cancer (NSCLC), which constitutes 85% of all LC cases [2] As an example, the three-year relative survival rate for NSCLC has improved significantly in the United States, rising from 26% for cases diagnosed in 2004 to 43% for those diagnosed in 2018 [3]. This positive trend is largely attributed to multiple factors, including stricter anti-smoking policies [4,5]; advancements in therapeutic strategies [6,7,8,9,10], especially the introduction of targeted therapies and immunotherapy [11,12,13,14]; and a diagnostic shift towards earlier-stage detection, facilitated by the increased use of chest CT scans [15,16] and screening programs [17,18,19].

Despite these advancements, real-world data indicate that LC survivors remain at high risk for cardiovascular disease (CVD) [20,21,22]. LC and CVD share common risk factors, and their combined burden may be further exacerbated by the widespread use of radiotherapy, chemotherapy, and immunotherapy.

This review aims to explore the cardiovascular (CV) implications of current NSCLC treatment strategies in real-world settings, with a particular focus on the frontline use of immunotherapy in adjuvant and neoadjuvant settings.

## 2. The Paradigm Shift of LC Management: Early Detection and Chemo-Immunotherapy

A key determinant of NSCLC prognosis is the stage at diagnosis. In recent years, the increased use of chest CT imaging, the development of algorithms for managing suspicious lung nodules, and the implementation of screening programs for high-risk individuals have contributed to a growing proportion of early-stage diagnoses [15,16,17,18,19,20,23,24,25,26]. However, the true breakthrough in NSCLC management has been the paradigm shift in treatment driven by immune checkpoint inhibition [12,13,14]. Targeting pathways such as programmed cell death protein 1 (PD-1/PD-L1) and cytotoxic T-lymphocyte-associated protein 4 (CTLA-4) has enabled the reactivation of the patient’s immune system to combat tumors, revolutionizing LC therapy.

Immune checkpoint inhibitor (ICI) impact on the immune system has been exploited in many settings:

(a) Metastatic Setting

The phase 3 KEYNOTE-024 trial (2016) demonstrated that pembrolizumab significantly improved progression-free survival (PFS) and 6-month overall survival (OS) compared to platinum-based chemotherapy in patients with metastatic NSCLC with high PD-L1 expression (≥50%) and no EGFR/ALK alterations [27]. A 5-year follow-up confirmed the durability of this benefit [28]. The survival advantage of immunotherapy over chemotherapy has been further validated in patients included in studies who received either alternative antibodies targeting PD-1 or agents directed against its ligand, PD-L1 [29,30,31,32]. The addition of anti-PD-L1 antibodies to platinum-based chemotherapy has significantly improved OS in metastatic, non-oncogene-addicted NSCLC, regardless of PD-L1 expression [33,34], and in NSCLC without EGFR/ALK/ROS1 alterations [35]. Chemotherapy reinforces the favorable result if associated with immunotherapy as first-line treatment [36]. Additionally, various ICI-based combinations have been explored with favorable results, including the addition of an anti-CTLA-4 or anti-VEGF agent [37,38]. Based on these data, chemo-immunotherapy (CIT) has been established as the standard of care in the first-line treatment of non-oncogene addicted metastatic NSCLC for patients with good performance status, any histology, and any PD-L1 expression [39].

(b) Locally Advanced, Unresectable NSCLC Setting

In the PACIFIC trial, durvalumab as consolidation therapy after chemoradiotherapy significantly prolonged PFS in patients with locally advanced, unresectable NSCLC [40]. ICIs have also been explored in combination with radiotherapy, where a synergistic effect with favorable microenvironment remodeling has been reported, although further studies are needed to optimize RT modality, timing, and ICI therapy duration [41].

(c) Perioperative Setting

Approximately 20–30% of patients with NSCLC present with resectable disease [42]. This proportion is expected to rise with the implementation of LC screening programs [43], which, however, will primarily target a population with a high prevalence of CV comorbidities [44]. Relapse rates remain high in resectable NSCLC despite the use of platinum-based neoadjuvant or adjuvant chemotherapy, which offers only modest (~5%) survival benefit [45,46]. As a result, there is growing interest in incorporating ICIs into perioperative treatment strategies [47].

In the neoadjuvant setting, the intact primary tumor may act as a source of neoantigens, stimulating tumor-specific T cells and enhancing systemic antitumor immunity [48]. The pivotal study by Forde et al. [49] demonstrated that neoadjuvant nivolumab plus chemotherapy significantly improved pathological complete response (PCR) and event-free survival (EFS) compared to chemotherapy alone in patients with stage IB–IIIA NSCLC. These findings have been supported by other trials [50,51,52] and incorporated into clinical guidelines, which now recommend neoadjuvant CIT (nCIT) followed by surgery as the standard of care [53]. However, concerns remain regarding nCIT-related toxicities in real-world practice, which may delay or preclude surgery, affect the extent and completeness of resection, and increase the likelihood of conversion from minimally invasive surgery to thoracotomy [49,50,51,52]. Adverse events and immune-related inflammation, including dense adhesions and fibrosis, are thought to contribute to these surgical challenges. To extend the survival benefit of nCIT and reduce the percentage of missed surgery, the role of an established multidisciplinary team (MDT) is of primary importance [54,55,56,57]. The CV implications of neoadjuvant cancer therapy are also increasingly recognized in Guidelines. The 2022 European Society of Cardiology (ESC) Guidelines on perioperative CV risk assessment identify “neoadjuvant cancer therapy” as a relevant risk factor requiring enhanced monitoring [58]. Similarly, ESC Cardio-Oncology Guidelines underscore the importance of neoadjuvant treatments in perioperative CV risk stratification [59]. Notwithstanding these caveats, neoadjuvant ICI therapy appears more effective than adjuvant therapy in both preclinical and clinical studies [60,61]. A meta-analysis of five neoadjuvant trials involving 2385 patients with stage II–III NSCLC compared neoadjuvant-only versus perioperative (neoadjuvant + adjuvant) ICI therapy. Adjuvant ICI did not improve EFS but was associated with a 14% higher incidence of grade ≥ 3 treatment-related adverse events (TRAEs) compared with chemotherapy alone, and an 8% increase in all-grade TRAEs relative to neoadjuvant-only ICI [62].

In the adjuvant setting, the IMPOWER-010 study showed an improvement in DFS in patients with resected NSCLC treated with atezolizumab after adjuvant chemotherapy compared with best supportive care (BSC) [63]. The final analysis of DFS and the second OS interim analysis after a follow-up ≥5 years confirmed the beneficial impact of adjuvant CIT [64]. Subsequent trials with both pembrolizumab and durvalumab provided conflicting evidence on the role of pure adjuvant immunotherapy [65,66]. Key phase 3 trials establishing ICI therapy in the perioperative NSCLC setting are summarized in Table 1 [49,50,51,63,64,65,67,68].

## 3. Lung Cancer Patients and Cardio-Immuno-Metabolic Risk

Longer survival exposes patients to competing causes of mortality, among which CVD is particularly relevant. CVD is highly prevalent in LC patients, affecting 30–50% of cases [69,70,71] and contributing to increased mortality (~30%) when coexisting with LC [22,72]. CVD is the second leading cause of death in LC patients, following cancer progression [73], and its impact on treatment decisions is well documented [74]. The rising trend of CVD-related hospitalizations among LC patients and the higher incidence of LC in individuals with pre-existing CVD [75,76] highlight a strong, bidirectional relationship between the two conditions. This link is further reinforced by shared risk factors, such as smoking and airborne environmental contaminants (AECs) [77]. Beyond traditional exposures, lifestyle-related CV risk factors—such as physical inactivity, unhealthy diet, and metabolic dysfunction—are increasingly recognized as contributors to LC risk. Active smokers often engage in poor lifestyle habits that adversely affect immuno-cardio-metabolic balance [78,79]. Sedentary behavior [80] and unhealthy dietary patterns [81,82,83,84] have also been associated with increased LC risk. In the UK Biobank cohort (416,588 participants, 1782 LC cases), a diet rich in fruits, vegetables, whole grains, and fiber—but low in red and processed meats—was linked to reduced LC incidence [85]. LC was more frequent in older males with lower socioeconomic status, higher smoking and alcohol use, and poorer education.

Metabolic syndrome (MetS), characterized by insulin resistance and elevated CV risk, has been independently associated with increased LC risk [86]. This association is supported by registry data [87,88], meta-analyses [89,90], and Mendelian randomization studies that suggest a causal link [91]. Genetic predisposition appears to mediate the influence of environmental and metabolic factors on LC development [92,93]. The traditional inverse relationship between body mass index (BMI) and LC prognosis [94]—often termed the “obesity paradox”—is now better understood as a limitation of BMI itself. The “obesity paradox” likely reflects that BMI does not distinguish between lean and fat mass, nor does it account for fat distribution. Higher BMI can indeed mask sarcopenia (a “BMI paradox”); patients with preserved BMI often have better nutritional reserves and performance status, which are favorable prognostic factors. Measures of central adiposity, such as waist circumference (WC), are more accurate indicators of metabolic health and LC risk. A meta-analysis of six prospective studies (831,535 participants, 5827 LC cases) found a 10% increase in LC risk per 10 cm increase in WC [95]. Similar findings from a pooled analysis of 12 global cohort studies (1.6 million participants, 23,732 LC cases) showed the highest risk in individuals with BMI < 25 kg/m^2^ but elevated WC [96]. This observation underscores the importance of body composition over BMI in predicting LC risk and outcomes [97]. Visceral adiposity and metabolic disturbances, including insulin resistance, may drive LC pathogenesis [98,99,100]. Sarcopenia—defined by reduced lean mass and often accompanied by increased visceral and ectopic fat—is found in ~50% of NSCLC patients [101] and is associated with chronic inflammation and accelerated CVD progression [102]. Skeletal muscle is involved in immune modulation [103,104] and insulin sensitivity [105], whereas excess visceral and intramuscular fat promotes inflammation, impairs muscle regeneration, and disrupts metabolic homeostasis [106]. This issue is particularly relevant in the era of immunotherapy as first-line treatment for NSCLC. Baseline sarcopenia during ICI therapy is linked to poorer treatment response and shorter survival [107,108]. Conversely, overweight or obese patients (by BMI) with preserved functional status (ECOG-PS 0–1), likely reflecting adequate muscle mass, tend to show better outcomes with ICIs [109]. Immune checkpoint proteins such as PD-1 and PD-L1 are expressed on the surface of T cells and have an important role not only in suppressing T-cell-driven inflammation but also in acting as negative regulators of atherosclerosis. Their inhibition, therefore, may induce accelerated atherosclerosis by increasing effector T cell responses and downgrading the atheroprotective regulatory T function, thus disrupting the delicate balance between T cell subsets [110,111,112,113,114]. Atherosclerosis can indeed be considered a “T-cell-driven disease”, as stated by Libby [115].

## 4. A Tale of Two Cities: Cardiovascular Toxicity of Immune Checkpoint Inhibitor Therapy in Randomized Clinical Trials Versus Real-World Practice

The design of randomized clinical trials (RCTs)—particularly inclusion/exclusion criteria and follow-up duration—significantly impacts their applicability to real-world clinical practice [116]. In pivotal RCTs of ICIs, patients with poor performance status, autoimmune conditions, or significant comorbidities such as established CVD were generally excluded or underrepresented [117,118]. Furthermore, key CV risk factors were often underreported [119]. Following reports of rare but fatal cases of fulminant myocarditis associated with ICIs [120], concerns have emerged regarding their CV safety, especially immune-related adverse events (irAEs). However, analyses of RCTs suggest that myocarditis and other immune-mediated CV events remain rare, with no significant increase in incidence [121,122,123,124,125]. Regarding CV safety, a systematic analysis of 63 RCTs including 32,518 patients (48 trials with control arms, 29,592 patients) identified an increased risk of six ICI-associated CV adverse events (CVAEs): myocarditis, pericardial disease, heart failure (HF), dyslipidemia, myocardial infarction (MI), and cerebral ischemia with incidence rates from 3.2 to 19.3 per 1000 patients, in a median follow-up of 3.2 to 32.8 months [126]. Pharmacovigilance data have further confirmed these associations. The 2018 WHO VigiBase study (31,321 patients) reported significantly elevated risk of ICI-induced myocarditis, pericardial disease, and vasculitis [127]. CVAEs occurred predominantly in men, often within one month of ICI initiation, and had fatal outcomes in over 80% of cases. Notably, myocarditis associated with ICI combination therapy had a higher mortality (65.6%) and was frequently accompanied by myositis and myasthenia gravis compared to myocarditis observed in ICI monotherapy (44.4%). Pericardial disease was more common in LC patients [127]. The rapid uptake of ICIs into clinical practice, driven by RCT success and reimbursement policies [128,129,130,131], has prompted increased awareness of irAEs [59,132,133]. Over time, CVAE profiles have evolved. For example, fatality rates for myocarditis have declined from 46% in 2018 [134] to 27.6% post-2020 [135]. Furthermore, real-world studies have broadened the spectrum of observed CVAEs. In a retrospective analysis by Jain et al. [136], ICI-treated patients with advanced cancers exhibited a 4.6% incidence of stroke, a 3.5% incidence of HF, a 2.1% incidence of atrial fibrillation (AF), a 1.5% incidence of conduction disorders, a 0.9% incidence of MI, a 0.05% incidence of myocarditis, a 0.05% incidence of vasculitis (0.05%), and a 0.2% incidence of pericarditis. A pharmacovigilance study by Cheng et al. [137], using the FDA Adverse Event Reporting System (FAERS) and The Cancer Genome Atlas (TCGA), identified older age, male sex, anti-PD-L1 agents, prior adverse events, and concurrent use of proton pump inhibitors, NSAIDs, or antibiotics as risk factors for severe CVAEs during anti-PD-1/PD-L1 therapy. Genetic and immune markers such as PD-L1 mRNA expression and LDL receptor-related protein 3 (LRP3) were also implicated. Severe CVAEs were fatal in over 30% of reported cases. While pharmacovigilance studies are valuable for detecting rare and late-onset ICI-associated CVAEs, but are limited by underreporting and selection bias. Clinical registries offer a more reliable real-world perspective. In a Danish national registry study (2011–2017), D’Souza et al. [138] evaluated the association between ICI therapy and CV events in LC and melanoma patients. Among 25,573 LC patients, 743 received PD-1 inhibitors (PD-1i), with a 1-year absolute risk of CV events of 9.7% (95% CI: 6.8–12.5). The hazard ratio (HR) of CV events was 2.14 (95% CI 1.50–3.05) within six months of ICI initiation and remained elevated (HR 2.26, 95% CI 1.27–4.02) beyond six months. Comparable findings emerged from a systematic review of 26 cohort studies (109,883 patients), which reported an 8.2% incidence of major adverse cardiac events (MACEs). ICI therapy, age, male sex, and prior radiation exposure were significantly associated with increased MACE risk [139]. A European multicenter study involving 1571 cancer patients (46.5% with LC) reported a 12.5% incidence of CVAEs—including HF, AF, MI, myocarditis, pericarditis, vasculitis, and Takotsubo cardiomyopathy—over a median follow-up of 8 months. CVAEs were more frequent and showed a later onset time compared to those reported in RCTs, highlighting the need for extended monitoring in clinical practice [140]. A recent meta-analysis of 15 observational studies (2019–2023) found that ICI therapy—either as monotherapy or in combination with chemotherapy—was associated with a significantly increased risk of cardiotoxicity compared to chemotherapy alone. The risk was especially pronounced in patients with pre-existing heart disease (OR 2.01; 95% CI: 1.64–2.46) and those with LC (OR 1.46; 95% CI: 1.26–1.69), with higher susceptibility observed in males, smokers, and older adults [141]. Real-world data have also elucidated a link between ICIs and chronic inflammatory processes such as atherosclerosis. ICIs can exacerbate pre-existing inflammation, and immune checkpoints are known to be negative regulators of atherosclerotic cardiovascular disease (ASCVD) in preclinical models [142,143,144]. Chronic inflammation, fueled by maladaptive immune responses and metabolic dysfunction, plays a central role in atherogenesis [145]. Clinically, ICI-induced “accelerated atherosclerosis” is increasingly documented [112,146,147,148,149]. Real-world evidence has also documented ICI efficacy and safety in underrepresented populations. Pasello et al. [150] observed that older adults and patients with poor performance status (PS), viral infections (e.g., HIV, hepatitis), or multiple comorbidities exhibited treatment outcomes comparable to those seen in RCTs, while the CheckMate 153 trial that included advanced NSCLC patients aged ≥ 70 with ECOG PS 2, demonstrated manageable safety and potential long-term survival benefits with nivolumab [151]. A retrospective analysis of 11,888 ICI-treated patients from the U.S. Veterans Affairs database—characterized by high comorbidity burden (Charlson Comorbidity Index [CCI] ≥ 3 in 68.9% of patients)—reported lower OS than RCTs, yet still superior to non-ICI therapies [152]. Similarly, Johns et al. found no association between specific comorbidities and irAEs or OS; however, a higher modified CCI was linked to shorter OS [153]. Hu et al. examined 125 patients with poor PS (ECOG ≥ 2: 35.2%), high CCI (≥3: 80.8%), autoimmune disease (12.8%), infections (11.2%), and brain metastases (20.8%). Despite their complex clinical profiles, these patients showed comparable treatment access, tolerability, and outcomes. Notably, white patients had shorter OS than non-white patients in multivariable analysis [154]. Regarding autoimmune disease, Lee et al. [155] demonstrated, in a matched cohort study (502 patients), that patients with pre-existing autoimmune conditions had nearly twice the risk of both CV and non-CV irAEs during ICI therapy. Finally, a decade after its introduction and widespread implementation in oncology, immunotherapy has emerged as a revolutionary treatment modality. However, its application in real-world clinical settings necessitates rigorous patient evaluation and meticulous monitoring.

## 5. Management of Cardiovascular Risk and Toxicity in NSCLC Patients Treated with Immune Checkpoint Inhibitors as First-Line Therapy

LC currently represents the most common indication for ICIs, with usage expected to rise due to expanding first-line indications and integration into both adjuvant and neoadjuvant settings. Consequently, managing ICI-related toxicities has become a key focus in cardio-oncology [156].

The real-world clinical complexity of LC patients, along with the emergence of chronic adverse events, presents ongoing challenges—particularly given that some studies suggest a potential association between immune-related adverse events and improved tumor response [157,158]. Preventing or at least mitigating CV complications is essential for treatment success and long-term outcomes, especially in the neoadjuvant setting where toxicity may compromise the timing and feasibility of surgical interventions and affect the postoperative course [57,159,160,161].

Real-world LC patients frequently present pre-existing structural heart disease and elevated CV risk [69,162,163,164,165], which significantly influence both therapeutic strategies and prognosis [71,163,166,167,168]. Moreover, in addition to immunotherapy, standard LC treatments such as surgery, thoracic radiotherapy (RT), and systemic chemotherapy also contribute to heightened CV risk and toxicity (Table 2 and Table 3).

Symptomatic post-RT cardiac events have an incidence rate of 11–32% [181,182]. Arrhythmias, CAD, and HF are the most frequent adverse CV events [171]; as a matter of fact, a few studies examine the different phenotypes of CV toxicities, and adverse CV events are mainly described as a sum of CV toxicities. Post-RT cardiac events are largely due to the peculiar vulnerability of lung cancer patients who have a heavy burden of comorbidities that enhance the potential CV toxicity of all oncologic therapies [171,183].

Several studies over the years have investigated strategies to stratify and predict the risk of CV complications in patients with NSCLC. A recent systematic review synthesized this evidence, identifying age, male sex, and advanced disease stage as relevant risk factors for acute CV events. Moreover, the presence of pre-existing CVD and the cumulative cardiac radiation dose were found to be the most significant predictors of long-term adverse CV outcomes [184].

Advanced age (>75 years), a high comorbidity burden, pre-existing CVD, and the use of concomitant chemotherapy have been identified as key factors associated with an increased risk of CV complications during immunotherapy [185,186,187]. Among comorbid conditions, beyond the potential impact of chronic obstructive pulmonary disease (COPD) on immune-related pneumonitis and its consequent effects on the cardiovascular system [188,189], type 2 diabetes mellitus has emerged as a significant negative prognostic factor. Its presence has been associated with both an increased risk of CV complications and a reduced therapeutic response to pembrolizumab-based immunotherapy [190,191]. Diabetes mellitus and its commonly associated metabolic conditions, such as central obesity and metabolic syndrome, are frequently characterized by dysregulation of immune-inflammatory responses and increased sympathetic nervous system activity (sympathetic overdrive) [192,193]. These alterations may contribute to an immunosuppressive microenvironment, potentially impairing the efficacy of ICIs and leading to reduced responsiveness to immunotherapy [194]. The alterations in the immuno-inflammatory and metabolic profile are further exacerbated by the presence of sarcopenia, often associated with systemic inflammation, metabolic dysregulation, and reduced physiological reserve, which may not only increase the risk of treatment-related toxicity and postoperative complications but also impair antitumor immune responses and reduce the likelihood of clinical benefit from ICI and chemotherapy tolerance [107,195] Surgical intervention is a major physiological stressor that exacerbates systemic inflammation. Thus, evaluating the patient’s baseline nutritional, metabolic, and inflammatory status is crucial, especially when planning neoadjuvant or adjuvant chemotherapy and/or immunotherapy, and considering potential CV implications.

In addition to evaluating functional capacity through measures such as the Eastern Cooperative Oncology Group Performance Status (ECOG-PS), the 6 min walk test, or cardiopulmonary exercise testing, biomarkers that specifically reflect inflammatory and immune status as preoperative C-reactive protein, interleukin-6, fibrinogen, and tumor necrosis factor-alpha, may prove useful in refining individual patient risk stratification [196]. In this context, indices derived from routine laboratory tests, such as the neutrophil-to-lymphocyte ratio (NLR) or the systemic immune-inflammation index (SII), appear potentially promising due to their repeatability and low cost [197,198,199,200,201]. Regarding the nutritional aspects associated with inflammation, promising preliminary data have emerged from the CALLY index, a novel, low-cost, and reproducible tool that incorporates serum albumin, C-reactive protein, and total leukocyte count to provide a composite measure of inflammatory-nutritional status [202,203]. However, these findings are based on retrospective registries with significant heterogeneity in both treatment approaches and patient pathologies and often rely on a single biomarker value. Prospective studies are thus warranted to confirm their definitive role in clinical practice.

The use of cardiac biomarkers, such as natriuretic peptides and particularly troponins, is recommended by the ESC cardio-oncology guidelines for the monitoring of potential cardiac adverse events associated with ICIs [59] Additionally, the European Society of Anesthesiology recommends the use of these biomarkers to enhance the predictive accuracy of the Revised Cardiac Risk Index (RCRI) for the prevention of postoperative cardiac events in patients undergoing non-cardiac surgery [204,205]. Basal preoperative troponin and natriuretic peptide (NP) evaluation in the context of LC surgery with planned additional treatments such as immunotherapy, chemotherapy, or radiotherapy is therefore mandatory for preoperative CV risk assessment and therapy adjustments in accordance with cardiologic international guidelines on CV management of patients undergoing non-cardiac surgery [58,206]. Detectable blood troponin levels have recognized prognostic value and improve CV risk stratification in the general population [207], and this is especially relevant in populations at risk for LC. In the prospective LUSI study [208], conducted in a cohort enrolled in a lung cancer screening program, 18% of patients without a history of CVD exhibited detectable troponin levels; in these patients, the risk of CV events and death increased proportionally with biomarker levels. The detectable blood level of troponin correlates with subclinical coronary atherosclerosis [209]. This condition is common and clinically significant in patients at risk for, or diagnosed with LC, as demonstrated by studies utilizing chest computed tomography (CT) [210,211,212,213,214,215,216], even when imaging is not ECG-synchronized [217]. In this context, the addition of information on coronary (and vascular) calcifications obtained from CT scans could further enhance individualized CV risk stratification and support the personalization of CV management strategies [218]. Scheduled serial chest CT scans performed during follow-up may also be helpful in assessing the progression of atherosclerosis in patients undergoing treatment with ICIs and/or chemotherapy [148,149]. Baseline multiparametric evaluation with biomarkers and CT imaging may be useful in the differential diagnosis with ICI-induced myocarditis [219]; the rapid increase in natriuretic peptides, troponin and CPK-MB associated with ventricular arrhythmias, the appearance of new conduction disturbances at the ECG, substantial deterioration of the ejection fraction are elements of suspicion for myocarditis, whose diagnosis, however, requires confirmation by cardiac nuclear magnetic resonance imaging [220]. Therefore, given the complex interplay of oncologic treatments and CV risk, multidisciplinary care is essential to ensure optimal CV risk stratification and management throughout the treatment continuum, with particular attention to baseline CV status and predisposing risk factors. A critical component of this approach is a comprehensive baseline CV assessment, which involves integrating clinical history, imaging studies, and laboratory biomarkers, and harmonizing these data with the overall oncologic treatment plan [59]. In this perspective, the involvement of cardiologists, aimed at optimal control of CV risk, can greatly help the multidisciplinary management in candidates for perioperative ICI treatment, especially if associated with chemotherapy [57,161,221].

Although most available data from earlier retrospective studies indicate that beta-blocker use has a neutral effect on survival in lung cancer patients [222,223], promising preliminary evidence suggests a potential therapeutic benefit in the setting of ICI therapy [224,225]. Tumor cells often express adrenergic receptors, and adrenergic signaling has been linked to cancer progression [226,227]. Thus, beta-blockers may enhance the efficacy of ICIs. However, current evidence is mostly retrospective, with some conflicting data [228] highlighting the need for proper patient selection and prospective studies to clarify their clinical utility.

Emerging observational data on the beneficial effects of sodium-glucose co-transporter 2 (SGLT2) inhibitors are encouraging, suggesting a potential role in reducing CV risk and enhancing the response to therapy [229,230,231]. Similarly, statins have shown promise not only in mitigating immunotherapy-related cardiotoxicity [141] but also in improving OS and PFS in patients receiving ICIs [232,233,234,235].

The current body of evidence supports a neutral association between GLP-1 RA use and LC risk [236], with some data suggesting a possible protective role [237,238]. However, these findings are primarily derived from retrospective studies in diabetic or obese populations. No direct evidence exists for a preventive or therapeutic effect in oncologic patients, especially those with lung cancer. Therefore, well-designed prospective studies are warranted to clarify the role of GLP-1 RAs in LC prevention and treatment.

### 5.1. Cardiovascular Events and Surgery in NSCLC

Surgical resection—including lobectomy, pneumonectomy, or sub-lobar resection—remains the standard of care for early-stage (stage I–II) and selected locally advanced (stage IIB–IIIA) NSCLC [53]. These procedures are frequently combined with perioperative chemotherapy and/or chemo-immunotherapy. Because surgical candidates generally have longer life expectancy, they also face a prolonged window of vulnerability to treatment-associated CV events. Thoracic surgery itself is burdened with a significant risk of CV complications, with reported rates of MACEs ranging from 6.5% to 22% [239,240,241]. Time-trend analyses highlight a growing prevalence of CV risk factors and increasing clinical complexity in surgical candidates, including ≥2 comorbidities [242]. Patients with pre-existing CVD and CV risk factors undergoing lobectomy or pneumonectomy are particularly vulnerable to postoperative CV complications [243,244,245,246].

Postoperative CV events may include arrhythmias, thromboembolic complications, MI, HF, stroke, or sudden cardiac death. However, determining the true incidence of specific postoperative CV events is complicated by the frequent aggregation of CV and respiratory complications in clinical reports, as well as inconsistent definitions of MACEs across studies [247].

Atrial fibrillation (AF) is the most common cardiac complication following LC thoracic surgery, with reported incidences as high as 30% [248,249,250], in the context of perioperative immunotherapy [54,161,251]. AF should not be underestimated due to its association with increased morbidity, including HF, stroke, and myocardial ischemia. The risk of AF is influenced by both the extent of surgical stress and patient-specific factors. AF is particularly prevalent after pneumonectomy, especially of the left lung [249,250,252]. Key risk factors include hypertension, obesity, advanced age, male sex, pre-existing CV or pulmonary disease, chronic obstructive pulmonary disease (COPD), tumor-related inflammation, and postoperative infections. Overall, patient frailty is also a critical determinant of AF risk [253,254,255]. Elevated natriuretic peptides or a history of previous AF can identify patients at higher risk of AF after major thoracic cancer surgery [256,257]. Postoperative atrial fibrillation (POAF) in non-cardiac surgery is linked to an increased long-term risk of stroke and mortality [258]. In a prospective, single-center study with continuous rhythm monitoring, approximately 30% of patients developed recurrent POAF one year after cancer surgery, most episodes being asymptomatic (92%). LC patients were at particularly high risk of relapses, with hypertension, renal dysfunction, and underlying structural heart disease emerging as independent predictors [259]. While the incidence of AF with ICIs appears low [141], individual risk assessment remains essential. Concomitant platinum-based chemotherapy and ancillary treatments, such as high-dose corticosteroids and NSAIDs, can predispose to arrhythmias [260]. Radiation therapy involving the cardiac base similarly elevates AF risk [171]. Moreover, AF may develop secondary to other ICI-related toxicities, including hyperthyroidism, pericarditis, or ICI-induced pneumonitis. The management of POAF requires special attention also for thromboembolic and hemorrhagic risk. In accordance with the 2024 AHA/ACC guidelines on noncardiac surgery [206], in patients who develop rapid AF intra- or postoperatively, it is reasonable to identify and correct precipitating factors (e.g., sepsis, anemia, pain) and, after carefully weighing the competing risks of thromboembolism and perioperative bleeding, to consider initiating postoperative anticoagulation. For patients with new-onset AF in the noncardiac surgical setting, outpatient follow-up is recommended to perform thorough thromboembolic risk stratification and ongoing AF surveillance, given the high likelihood of recurrence and evolution in permanent AF. These recommendations echo current AF guidelines [261,262] by emphasizing the need for a multidisciplinary team involving cardiologists to optimize both cancer and AF management while minimizing drug–drug interactions, QTc prolongation, proarrhythmic risk, bleeding, and thromboembolism. Arrhythmia management should be individualized according to hemodynamic stability and overall clinical status. POAF is transitory with no clear superiority of rate-control versus rhythm-control strategy in this setting. Prophylactic β-blockade as a unique indication for POAF prevention in noncardiac surgery is not recommended, as it has been associated with increased mortality [262,263]. However, as sympathetic overdrive precipitates postoperative tachyarrhythmia, in the absence of contraindications, β-blockers remain the first-line therapy. In cases of contraindications or relative β-blocker resistance, combination regimen or alternative agents such as Digitalis or non-dihydropyridine Ca-channel blockers may be employed. Amiodarone, while effective [261], carries risks of thyroid dysfunction and acute pulmonary injury complications when high dosages are administered [264]. Furthermore, these complications may overlap with immune checkpoint inhibitor–related toxicities [265]; therefore, its use in pulmonary resection on perioperative ICI therapy warrants extreme caution. For most cancer patients with AF, whether in remission or on active treatment, direct oral anticoagulants (DOACs) are preferred over vitamin K antagonists for stroke prevention and bleeding [261,266,267]. This choice is further supported by evidence that ICI therapy is associated with an increased risk of thromboembolic complications [141]. In patients with nonvalvular atrial fibrillation receiving non-vitamin K oral anticoagulants, concomitant use of amiodarone, digoxin, diltiazem, verapamil, and antidepressants may be harmful [268,269] and should be approached with caution and dynamic individualized assessment.

Peri-operative myocardial injury and troponin elevation occur in approximately 20% of patients undergoing thoracic surgery [270]; in the current series of patients undergoing elective LC surgery, even higher incidences are reported, up to 49% [271,272]. Elevated troponin levels above the 99th percentile reference value in the postoperative period following non-cardiac surgery are indicative of cardiac stress or injury/infarction and are strong predictors of both short- and long-term mortality and morbidity [273]. Accordingly, current international guidelines for CV risk assessment and management of non-cardiac surgery recommend routine troponin monitoring in moderate–high risk patients [58,206]. Troponin elevation in the surgical population may vary according to age, sex, renal function, as well as type and urgency of the surgery and does not necessarily indicate a postoperative MI caused by coronary plaque rupture or thrombosis (i.e., Type 1 MI according to Thygesen) [274] in the absence of other clinical signs such as symptoms, ECG changes, or evidence of atherosclerotic coronary obstruction. Type 1 MI is relatively rare (approximately 1%) in the setting of thoracic surgery, especially with the use of current minimally invasive techniques [275,276]. In the absence of secondary causes such as hypoxemia, hypotension, anemia, HF, pulmonary embolism, or arrhythmias and infections, troponin elevation is typically attributed to an imbalance between myocardial oxygen supply and demand (Type 2 MI) [274,277]. Troponin elevation generally occurs early, within 72 h postoperatively, and is frequently asymptomatic. Nonetheless, it carries a significant negative prognostic impact on both short- and long-term mortality and morbidity. Therefore, patients with this condition require close monitoring and aggressive management of factors sustaining myocardial injury.

### 5.2. Pericarditis

Pericardial disease is a relevant clinical issue in lung cancer (LC) patients and may be presented as acute pericarditis, pericardial effusion, or constrictive pericarditis. In LC, pericarditis can result from direct pericardial involvement by the primary tumor, metastatic spread, or prior thoracic radiotherapy [278,279]. Cases of ICI-associated pericarditis have also been described [136,140,280]. The occurrence of pericarditis in LC patients receiving ICIs is particularly challenging, as its etiology can be multifactorial and often difficult to determine. Management of acute pericarditis generally includes nonsteroidal anti-inflammatory drugs (NSAIDs) and colchicine, with corticosteroids or anti-IL-1 agents reserved for refractory cases [279]. For pericardial effusion, pericardiocentesis, prolonged catheter drainage, or a pericardial window may be required, while pericardiectomy may be indicated for radiotherapy-induced constrictive pericarditis [279].

## 6. Survivorship and Cardiovascular Surveillance

Improved survival in LC patients presents major challenges for survivorship care, requiring a framework that extends beyond oncologic follow-up to encompass psychosocial support and the management of treatment-related complications [281]. LC survivors face a particularly elevated risk of CV events, which emerges earlier than in other malignancies [21,282,283] and may be further exacerbated by immune checkpoint inhibitor (ICI) therapy [140,284]. In NSCLC, this issue is compounded by substantial heterogeneity related to tumor type and stage, therapeutic protocols, and the high burden of comorbidities, as many patients are elderly, at elevated CV risk, or have pre-existing heart disease. With the number of survivors projected to rise substantially in the coming decade [281,285], there is an urgent need for comprehensive strategies integrating CV risk assessment, prevention, and long-term management. However, available evidence—largely from retrospective analyses of RCTs and non-dedicated registries—provides limited guidance for preventive strategies. Historically, research has focused on immune-related myocarditis, while only recently has growing scientific interest expanded and quantified the broader spectrum of CV complications. Furthermore, the characterization of chronic complications such as atherosclerosis remains incomplete and warrants further investigation.

Traditional CV risk factors—hypercholesterolemia, hypertension, and obesity—are key determinants of atherosclerotic “cardiotoxicity” [286] and require careful management in patients undergoing ICI therapy. Supporting this, a multicenter retrospective study reported a 35% higher relative risk of incident hypertension in patients receiving ICIs compared with matched controls [287]. Similarly, a meta-analysis of 48 RCTs identified dyslipidemia as one of the most frequent CV complications in ICI-treated patients [126], while data from Drobni et al. suggested that concomitant statin therapy slows the progression of atherosclerotic plaques [147]. Beyond lipid metabolism, in NSCLC patients aged ≥ 66 years with pre-existing diabetes, SGLT2 inhibitor use was associated with reduced mortality (HR 0.68), with an even greater benefit observed with longer treatment duration (HR 0.54) in the SEER-Medicare cohort, suggesting that therapy initiation should not be delayed [230].

Epidemiological data—including Life’s Simple 7 and Life’s Essential 8 studies [288,289] and findings from the UK Biobank [290]—consistently demonstrate that optimal CV risk factor control improves long-term outcomes in cancer survivors. Yet, in clinical practice, risk factor management remains largely suboptimal [291,292]. Effective survivorship care must therefore address both medical and psychosocial determinants. Anxiety, depression, and cancer-related stigma are highly prevalent in this population [281,293] and may compromise adherence, social functioning, and even immune competence, potentially reducing responsiveness to ICIs [294]. All the components of emotional distress should be actively searched for [295].

Prevention strategies should include lifestyle interventions and structured rehabilitation. The cardio-oncology rehabilitation (CORE) model, proposed by Gilchrist in 2019, integrates exercise, nutritional counseling, and cardiovascular risk management, offering a comprehensive approach to reduce CV events [296]. This is particularly relevant for lung cancer patients, who often manifest reduced exercise tolerance and impaired quality of life, making both prehabilitation and rehabilitation beneficial [297]. Prehabilitation, particularly, improves functional capacity, reduces postoperative complications, and shortens hospital stay [298,299], and it is currently recommended by ASCO guidelines [300].

## 7. Conclusions

Immunotherapy has revolutionized lung cancer treatment, demonstrating a significant survival benefit, albeit at the cost of adverse events, among which CV complications stand out for their severity and clinical relevance. LC patients represent a complex cardio-immuno-metabolic population, often experiencing functional limitations that impair health-related quality of life (HRQoL) [293,301]. Managing individual lung cancer patients can be challenging due to the complex interplay of multiple variables. Nevertheless, this is an active area of research. For instance, microRNA biomarkers, which are more directly linked to the underlying pathophysiology, may become relevant for cardiotoxicity screening [302]. Additionally, several inflammatory chemokines [303] and interleukins (ILs), including IL-6, IL-8, and IL-10, involved in perioperative immune modulation, may help predict complications following lung cancer surgery [304,305]. Direct inhibition of IL-6 appears to have the potential to enhance the efficacy of immunotherapy while reducing the risk of adverse events [306,307], and its role in atherosclerotic coronary disease is currently under investigation [308]. Significant potential improvements are also anticipated in the field of imaging, such as the analysis of coronary plaques using coronary CT [309,310] and the assessment of their inflammatory profile with PET-CT [311,312,313].

In conclusion, there are three pillars of an integrated dynamic immuno-cardio-metabolic approach in LC patients in immunotherapy: (1) the risk stratification/awareness pillar that assesses (at baseline and throughout the cancer continuum) the CV risk score, the comorbidities and the metabolic-immuno-inflammatory profile through the evaluation of both metabolic, immune and inflammatory markers and imaging tools; (2) the multidisciplinary intervention that aims to reduce CV risk through preventive strategies and an aggressive control of CV risk factors; (3) the continuous multidisciplinary surveillance plan based on biomarker- and imaging-based monitoring. This plan has to include the assessment of the allostatic load, which is “*the price of adaptation to potentially stressful challenges*” or a failure to maintain “allostasis”, which is “*stability through change*” [314,315].

The valuable contribution of artificial intelligence is expected to support the main goal of management: delivering precise, tailored treatment across the entire cancer continuum, extending into the long-term survivorship phase [316,317,318,319].

We are, however, only at the beginning of a new era in the management of oncology patients, with growing awareness that social, economic, and organizational factors, by affecting equity and generalizability of care, may compromise treatment outcomes [320,321,322,323,324]. What is clear is that the management of these complex patients necessarily requires a multidisciplinary approach, in which the cardiologist must play an integral role and can no longer be excluded throughout the cancer journey.

## Figures and Tables

**Table 1:** Key studies in perioperative, neoadjuvant, and adjuvant settings.

Perioperative Trials							
Study/Ref	Treatment	n	Surgery	R0 Resection	pCR	EFS HR	OS HR
Checkmate 77T [67]	Nivo vs. Pbo + PBC	229 232	77.7% 76.7%	89.3% 90.4%	25.3% 4.7%	0.58 (0.42–0.81) *p* < 0.001	-
KEYNOTE-671 [51]	Pembro vs. Pbo + PBC	397 400	82.1% 79.4%	92% 84.2%	18.1% 4%	0.58 (0.46–0.72) *p* < 0.001)	0.72 (0.56–0.93) *p* = 0.0052
AEGEAN [50]	Durva vs. Pbo + PBC	366 374	77.6% 76.6%	94.7% 91.3%	17.2% 4.3%	0.68 (0.53–0.88) *p* = 0.004	-
Neoadjuvant trials							
Study	Treatment	n	Surgery	R0 resection	pCR	EFS HR	OS HR
Checkmate 816 (1st study) [49] Checkmate 816 (final analysis) [68]	Nivo vs. Pbo + PBC Nivo vs. Pbo + PBC	179 179 179 179	83.2 75.4	83.2% 77.8%	24% 2.2%	0.63 (0.43–0.91) *p* = 0.005	0.57 (0.30–1.07) *p* = 0.008 * 0.72 (95% CI 0.523 to 0.998, *p* = 0.048
Adjuvant trials							
Study	Treatment	n	DFS HR (ITT)		DFS HR (PD-L1 ≥ 50%)	OS HR (ITT)	OS HR (PDL1 ≥ 50)
IMPOWER-010 [63] IMPOWER-010 (DFS final analysis, 2nd OS interim analysis) [64]	PBC → Atezo vs. BSC PBC → Atezo vs. BSC	442 § 440 § 442 § 440 §	0.81 (0.67–0.99) *p* = 0.040 * 0.85 (0.71–1.01) *p* = 0.07 *		0.43 (0.27–0.68) 0.48 (0.32–0.72)	0.995 (0.78–1.28) 0.97 (0.78–1.22)	0.43 (0.24–0.78) *p* = 0.005 0.47 (0.28–0.77)
KEYNOTE-091 [65]	Optional PBC → Pembro vs. Pbo	590 587	0.76 (0.63–0.91) *p* = 0.0014		0.82 (0.57–1.18)	0.87 **	-

* did not cross the boundary for statistical significance; ** Immature data. § In both the IMPOWER 010 initial study and in the final analysis, 442 patients were treated with adjuvant PBC followed by atezo and 440 with BSC. Atezo: atezolizumab; BSC: best supportive care; DFS: disease-free survival; Durva: durvalumab; EFS: event-free survival; HR: hazard ratio; n: number of patients; Nivo: nivolumab; OS: overall survival; Pembro: pembrolizumab; PBC: platinum-based chemotherapy; Pbo: placebo.

**Table 2:** Chemo-radio lung cancer therapies associated with ICIs and their risk of both acute and long-term cardiovascular toxicities.

Agent	Cardiovascular Toxicity	Gaps in Knowledge
Chest radiation [169,170,171,172,173]	Pericardial effusion Atrial arrhythmias HF MI, CAD Conduction abnormalities Constrictive pericarditis	Timing of ICI administrations: Delayed PD-1/PD-L1 inhibitors administration after chest RT may lower early immune-related toxicities while preserving clinical benefit in selected patients. Indications: there are still limitations in elderly, multimorbid patients, and those with structural heart disease. RT-induced cardiovascular damage is still underestimated; cardiovascular toxicity of chest radiation + ICIs is not well defined. These concerns dictate an individualized surveillance and intensive risk factor control. Timing and targets of cardiovascular risk factors are not completely defined. Lipid-lowering therapies show promising effects in reducing CAD risk, but further validation is required.
Platinum-based therapies [174,175,176]	Hypertension Venous/arterial Thrombosis Long-term risk for CAD Atrial arrhythmias	Cardiotoxicity of platinum + immunotherapy in first-line lung cancer is understudied in real-world practice. Mechanisms of cardiovascular toxicity are not precisely defined yet. Patient risk stratification: individuals with pre-existing cardiovascular disease appear at higher risk, although sporadic events have also been reported in patients without risk factors. The optimal cardio-oncology surveillance and proactive risk factor management are yet to be defined.
Taxanes [59]	Atrial arrhythmias Transient sinus bradycardia Conduction disturbances (AV nodal blocks, LBBB) Increased thromboembolic risk	Current data suggest that the combination with ICI does not significantly increase cardiotoxicity compared with taxanes alone, but real-world evidence is limited. The modality of cardiac monitoring is yet to be defined in patients with preexisting cardiovascular disease.
Pemetrexed [177]	Peripheral edema	Pemetrexed is generally safe, but preexisting cardiovascular disease can be a liability. Modalities of cardiac monitoring have not been defined in this population.
Gemcitabine [178]	Venous/arterial Thrombosis CAD Atrial arrhythmias	Monitoring is recommended only in high-risk patients, but adverse cardiovascular events may occur in patients with no risk factors. Surveillance modalities are not well defined for patients treated with gemcitabine and ICIs
Vinorelbine [179]	HF CAD, arrhythmias	Sporadic cases of vinorelbine-induced bradycardia, supraventricular arrhythmias, or ischemic events are reported, mainly in patients with preexisting cardiovascular disease or when used in combination with other cardiotoxic drugs. Modalities of cardiac monitoring when vinorelbine is administered with ICIs have not been defined.
Etoposide [180,181]	HF CAD	Sporadic adverse cardiovascular events are reported mainly in patients with preexisting cardiovascular disease. Modalities of cardiac monitoring when etoposide is administered with ICIs have not been defined.

Abbreviations: AV: atrioventricular; CAD: coronary artery disease; HF: heart failure; ICIs: immune checkpoint inhibitors; LBBB: left bundle branch block; MI: myocardial infarction; RT: radiotherapy.

**Table 3:** (Summary table): Phenotypes of cardiovasculotoxicity of oncologic treatments associated with ICIs: chemotherapy (PBC, taxanes, pemetrexed, gemcitabine, vinorelbine, and etoposide) and chest radiotherapy (RT).

Arrhythmias/ Conduction Abnormalities	CAD	HF	Hypertension	Venous/Arterial Thrombosis	Peripheral Edema	Pericardial Disease
Chest RT PBC Taxanes	Chest RT PBC Gemcitabine Vinorelbine Etoposide	Chest RT# PBC Vinorelbine Etoposide	PBC	PBC Taxanes Gemcitabine	Pemetrexed	Chest RT

CAD: coronary artery disease; HF: heart failure; ICIs: immune checkpoint inhibitors; PBC: platinum-based chemotherapy; RT: radiotherapy.

## Abbreviations

The following abbreviations are used in this manuscript: AF: atrial fibrillation; ALK: anaplastic lymphoma kinase; BMI: body mass index; CAD: coronary artery disease; CALLY Index: C-reactive protein-albumin-lymphocyte index; CIT: chemo-immunotherapy; CKD: chronic kidney disease; COPD: chronic obstructive pulmonary disease; CT: computed tomography; CTLA-4: cytotoxic T-lymphocyte-associated protein 4; CV: cardiovascular; CVA: cardiovascular adverse events; CVD: cardiovascular disease; DM: diabetes mellitus; ECOG PS: Eastern Cooperative Oncology Group performance status; EGFR: epidermal growth factor receptor; ESC: European Society of Cardiology; FAERS: FDA Adverse Event Reporting System; GLP1 RA: glucagon-like peptide-1 receptor agonist; HbA1c: glycated haemoglobin A1c; hs-CRP: High Sensitivity C-reactive Protein; ICI: immune checkpoint inhibitor; IL-6: Interleukin-6; irAEs: immune-related adverse events; MI: myocardial infarction; LC: lung cancer; MDT: multidisciplinary team; MRI: magnetic resonance imaging; NLR: neutrophil-to-lymphocyte ratio; NP: natriuretic peptide; NSCLC: non-small cell lung cancer; OS: overall survival; PD-1/PD-L1: programmed cell death protein 1; PFS: progression-free survival; RCRI: Revised Cardiac Risk Index; RCT: randomized controlled trial; ROS1: ROS proto-oncogene 1; SGLT2: sodium glucose co-transporter 2; TCGA: The Cancer Genome Atlas; TyG Index: triglyceride-glucose index; SII: Systemic Immune-inflammation Index; TRAEs: treatment-related adverse events WBC white blood cells; WC: waist circumference.
